# A case report of a solitary pancreatic metastasis of an adrenocortical carcinoma

**DOI:** 10.1186/s12893-015-0076-3

**Published:** 2015-07-31

**Authors:** Johannes Baur, Ulla Schedelbeck, Alina Pulzer, Christina Bluemel, Vanessa Wild, Martin Fassnacht, U. Steger

**Affiliations:** Department of General, Visceral, Vascular and Pediatric Surgery, University Hospital Wuerzburg, Wuerzburg, Germany; Institute of Radiology, University Hospital Wuerzburg, Wuerzburg, Germany; Department of Nuclear Medicine, University Hospital Wuerzburg, Wuerzburg, Germany; Department of Internal Medicine I, Endocrinology, University Hospital Wuerzburg, Wuerzburg, Germany; Comprehensive Cancer Center Mainfranken, University of Wuerzburg, Wuerzburg, Germany; Institute of Pathology, University Wuerzburg, Wuerzburg, Germany

**Keywords:** Adrenocortical Carcinoma, Metastases to pancreas, Surgical treatment

## Abstract

**Background:**

Solitary metastases to the pancreas are rare. Therefore the value of resection in curative intention remains unclear. In the literature there are several promising reports about resection of solitary metastasis to the pancreas mainly of renal origin.

**Case presentation:**

Here we report for the first time on the surgical therapy of a 1.5 cm solitary pancreatic metastasis of an adrenocortical carcinoma. The metastasis occurred almost 6 years after resection of the primary tumor. A partial pancreatoduodenectomy was performed and postoperatively adjuvant mitotane treatment was initiated. During the follow-up of 3 years after surgery no evidence of tumor recurrence occurred.

**Conclusion:**

Resection of pancreatic tumors should be considered, even if the mass is suspicious for metastatic disease including recurrence of adrenocortical cancer.

## Background

Adrenocortical carcinoma (ACC) is a rare malignant tumor with an estimated incidence of 1 to 2 cases per million population [1]. The occurrence has a first peak in childhood and a second in the fourth and fifth decade of life. The majority of ACC are sporadic. But in some cases ACC is part of hereditary syndromes like Li-Fraumeni syndrome, Beckwith-Wiedeman syndrome, multiple endocrine neoplasia (MEN) 1, congenital adrenal hyperplasia or familial polyposis coli [2]. Therapy and prognosis strongly depend on the European Network for the Study of Adrenal Tumours (ENSAT) classification [3]. In localized disease, surgery is treatment of choice [4], whereas in advanced disease mitotane alone or in combination with cytotoxic drugs is standard of care [5]. According to the German ACC register, 5-year-survival rates are 84 %, 63 %, 51 % and 15 % for Stage I, II, III and IV respectively [6]. Management of recurrent disease is not standardized [5], but recent retrospective studies suggest that surgery is of benefit in selected cases [7].

Metastatic involvement of pancreas due to any primary malignant tumor is rare and represents about 2 % of all pancreatic tumors. Most of these patients show diffuse distant metastases of their primary tumor [8] with no opportunity of surgical treatment.

Here, we present a case of recurrent ACC with a solitary pancreatic metastasis 6 years after resection of the primary tumor.

## Case presentation

**Fig. 1:**
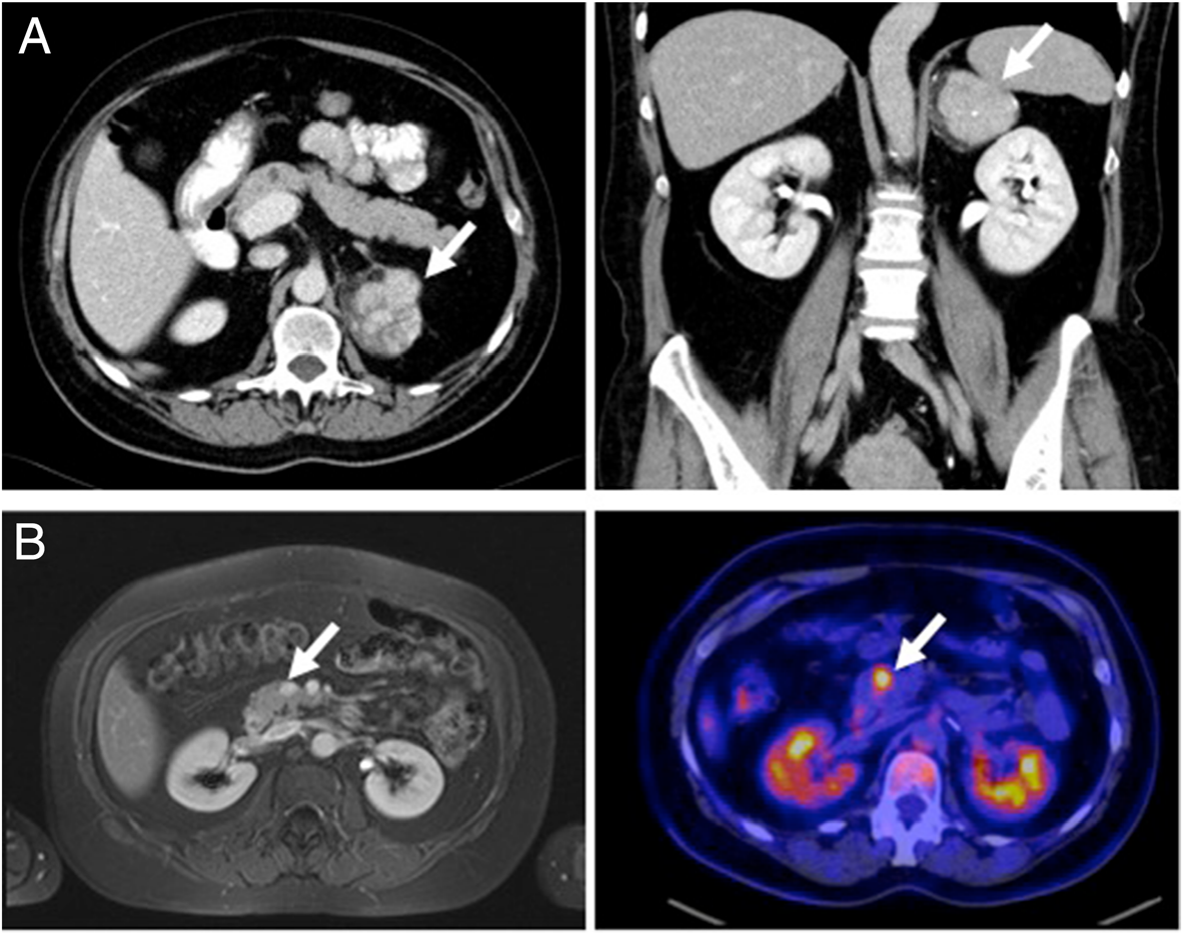
Imaging of the primary adrenocortical carcinoma (**a**) and the solitary metachronous metastasis inside the pancreatic head (**b**) by MRT and FDG-PET/CT, respectively

A 45-year-old woman was first diagnosed in 2006 with ACC showing typical signs of Cushing’s syndrome with consecutive increase of weight and body hair, acne, therapy-refractory arterial hypertension and decrease of physical working capacity. Hormonal work-up revealed elevated cortisol levels basal and after dexamethasone suppression, as well as elevated dehydroepiandrosterone sulphate (DHEA-S) and androgen levels. CT scan showed a 4.7 × 5.2 × 4.5 cm sized mass of the left adrenal gland with venous contrast enhancement suspicious for adrenocortical carcinoma (Fig. 1).

The patient underwent conventional resection of the left adrenal gland and lymphadenectomy. Pathological study of the resected tissue was performed by two independent pathologists. The tumor presented a solid growth pattern with some fibrous bands and small areas of necrosis. Tumor cells appeared monomorphic with small nuclei and condensated chromatin. The rate of mitosis was under 1 in 10 high power fields. Interestingly there were large amounts of myelolipomatosis metaplastic areas. All three classic scoring systems showed results still within the value range for benign lesions. However, a high Ki-67 expression of 20 % led to the diagnosis of an adrenocortical carcinoma (ENSAT stage II). Due to a good differentiation, no adjuvant mitotane therapy has been performed. Though the patient underwent follow-up examinations periodically, including hormonal work-up and CT or MR imaging performed initially every 3 months and eventually every 6 months after a 2-year-recurrence-free survival.

Sixty-six months after surgical treatment MR imaging showed a new hyperperfused mass in the head of the pancreas with progression of size in the following studies as well as a high FDG-uptake in positron emission tomography (Fig. 1). Thus, the mass was highly suspicious of malignancy and resection was recommended by interdisciplinary consensus.

The patient underwent pylorus preserving partial pancreaticoduodenectomy. Postoperatively the patient developed a pancreatic fistula with an intraabdominal abscess formation, which had to be drained interventionally on day 10 after surgery. The drain could be removed on day 24 after surgery.

**Fig. 2:**
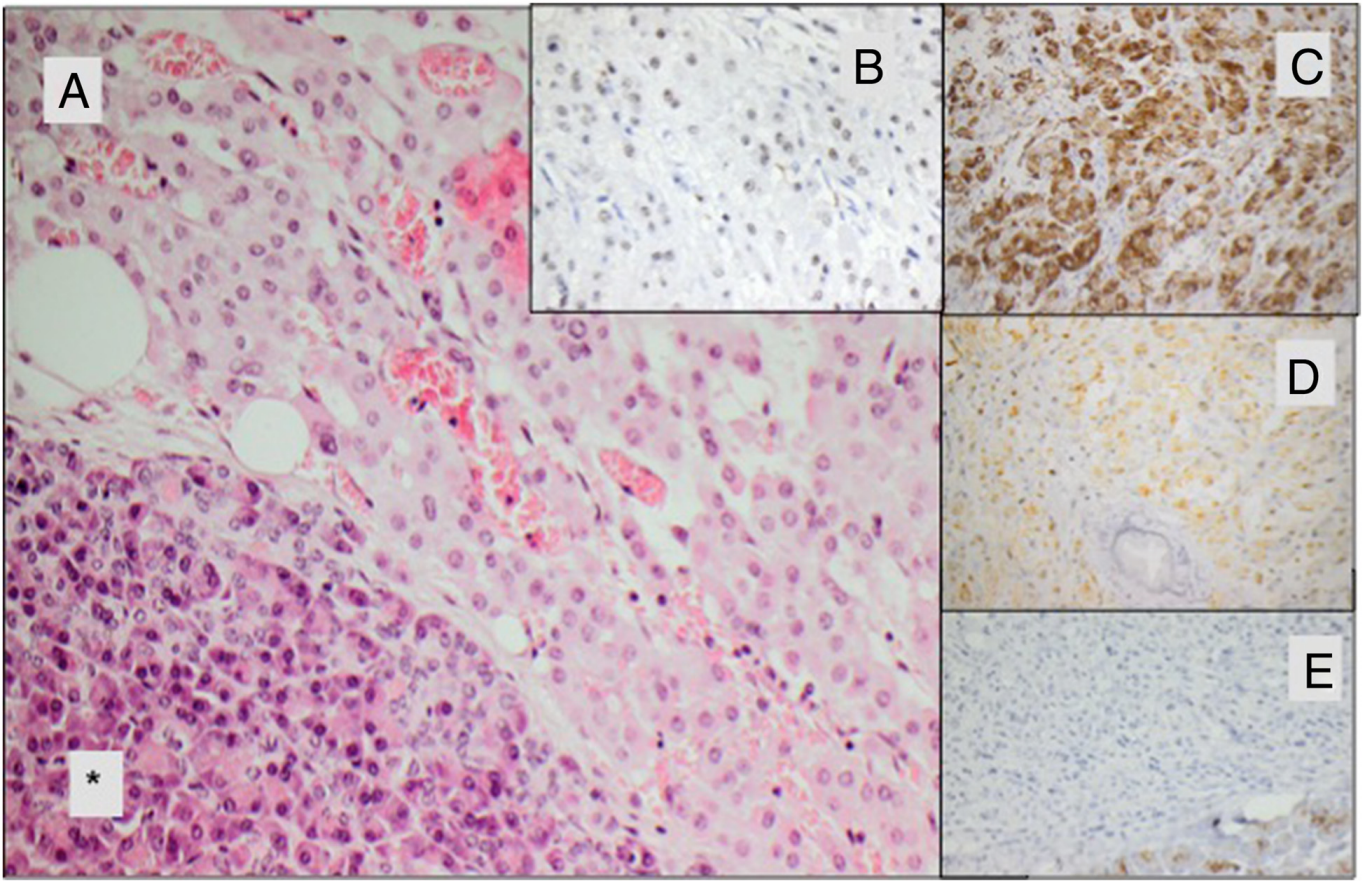
Histology of pancreatic mass (**a**) metastasis with adjacent pancreatic tissue (*) (HE stain, 20x) (**b**) ACC metastasis with nuclear positive staining pattern for SF-1 (SF1, 40x) (**c**) ACC metastasis with positive cytoplasmic staining for Synaptophysin (**d**) ACC metastasis with positive cytoplasmic staining for Inhibin [E] ACC metastasis is negative for pan-ck (AE1/3, 20x), the pancreatic acini stain positive for pan-ck

Histological and immunohistochemical examination of the resected tissue showed a 1.5 cm sized well differentiated tumor with solid growth pattern (Fig. 2a). Tumor cells presented monomorphic with eosinophilic cytoplasm and unremarkable nuclei. The metastasis stained positive for synapthophysin, Melan A and steroidogenic factor 1 (SF-1) in consistency with the diagnosis as a metastasis of the adrenocortical carcinoma (Fig. 2b) with a Ki-67 expression in 10 % of tumor cells. There where no signs of a primary pancreatic carcinoma as the tumor cells stained negative for pan cytokeratine (pan-ck) (Fig. 2c). In addition, pathological examination by the reference pathologist of the German ACC study group was performed and confirmed the correlation between the ACC primary tumor and the unusual site of metastasis.

According to interdisciplinary consensus an adjuvant mitotane therapy was initiated after the tumor resection and is still ongoing. During follow-up, including endocrine and imaging work-up every 3 months now for more than 3 years, there is no evidence of recurrence (Time axis see Table 1).

**Table 1:** Time axis

2006	Conventional left-side Adrenalectomie of an Adrenocortical Carcinoma (stadium II, diameter 7 cm)
2006 - 2012	Unsuspicious follow-up in periodically performed cross-sectional studies
02/2012	Detection of a pancreatic mass suspicious for malignancy in PET
03/2012	Pylorus preserving partial pancreaticoduodenectomy Histological and immunhistochemic examination showed a 1.5 cm sized well differentiated metastasis of the adrenocortical carcinoma
Till present date	Mitotane Therapy, unsuspicious follow-up in periodically performed cross-sectional studies and hormonal work-up

### Literature review

A literature review has been performed to identify the distribution of different tumor entities of solitary metastases inside the pancreas. A PubMed search for studies or case reports dealing with “metastases to pancreas” was performed. Articles were included in this review if 10 or more patients were investigated retrospectively or prospectively, patients suffered of metastases to pancreas (no infiltrative involvement) and only the pancreas was affected by metastases, patients received resection of pancreas metastases in curative intension and different tumor entities were included in each study. Four retrospective studies [9–12] summerized in Table 2 met the inclusion criteria with a total of 92 patients. Median age of patients ranged from 59 to 64 years. Most frequent origin of pancreatic metastases was the kidney in 46 % of the cases followed by melanoma in about 10 % of the cases. Rectal and Colon carcinomas were involved in only 5 cases. One “non-pancreatic endocrine tumor” was reported but not specified in more detail [12]. Here, the distribution of metastatic origin is different to other literature reviews, where the proportion of renal cell carcinomas range between 60 and 70 % [8]. This is caused by the exclusion of studies intending to investigate a single tumor entity metastatic to the pancreas (mostly renal cell carcinomas). Median survival after curative resection of pancreatic metastases was 2.2 to 4.3 years. 5-year-survival ranged between 36 % and 61 %.

**Table 2:** Literature review

Author (Year)	Years observed	No. of patients	Median age	Median survival (y)	5-year- survival (y)	RCC	Melanoma	Gall bladder	Sarcoma	Colon	Ovar	Lung	Breast	Others^a^
Reddy el al. (2008) [10]	1970–2007	49	60	3.7	36 %	21 (42.9)	3(6.1)	6 (12.2)	4 (8.2)	2 (4.1)	4(8.1)	4 (8.2)	1 (2.0)	4(8.1)
Bahra ct al. (2008) [7]	1989–2007	20	62	not reached	61 %	9 (45.0)	1 (5.0)	1 (5.0)	2 (10 .0)	1 (5.0)	-	-	-	6(12.2)
Eidt ct al. (2007) [9]	1993–2005	12	64	4.3	not reported	7(58.3)	4(33.1)	-	-	1 (8.3)	-	-	-	-
Crippa ct al. (2006) [8]	1994–2005	11	59	2.2	48 %	5 (45.5)	1(9.1)	-	-	1 (9.1)	1(9.1)	-	3 (27.3)	-
		92				42(45.7)	9.(9.8)	7(7.6)	6(6.5)	5(5.4)		4(4.3)	4(4.3)	10(10.9)

*RCC* Renal cell careinoma

^a^Schwannoma (reported twice); Seminoma, Teratocarcinoma, Hepatocellular Carcinoma, Langerhans cell histiocytosis, esophagus, mesechymal gastric tumor, non-pancreatic endocrine tumor (not specified), GIST (each one case reported)

## Discussion

Solitary metastases to the pancreas are rare. Only 1.3 % of patients undergoing pancreatic resection present a solitary metastasis of a primary solid tumor [12]. In our literature review singular metastasis to the pancreas was most frequent of renal origin (46 % of cases). The value of resection in curative intention remains unclear. As our literature review revealed, 5-year-survival may range between 36 % and 61 % if different tumor entities are taken together. Certain metastatic tumor entities even show better survival rates after curative resection compared to primary pancreatic carcinomas. In case of a singular pancreatic metastasis of renal cell carcinoma a 5-year-survival between 66 % and 79 % can be achieved by curative resection of pancreatic metastasis [8, 13].

Here we report for the first time a case of a solitary pancreatic metastasis of an adrenocortical carcinoma. ACC is one of the most aggressive endocrine tumors known so far. Resection of recurrent tumor is recommended in selected case (e.g. when the disease-free interval exceeds 12 months and complete resection seems to be feasible) [7]. In our case, the metastasis occurred almost 6 years after resection of a very well differentiated primary tumor and a R0 resection could be performed. Although known to improve recurrence-free survival after resection of primary tumor [14], it remains unclear if adjuvant mitotane therapy would have prevented recurrence of ACC. However, the patient would have suffered from mitotane side effects like adrenocortical insufficiency. As the patient is now again free of disease for more than 3 years, surgical approach to treat solitary pancreatic ACC metastasis was most likely of great benefit to her.

## Conclusion

In conclusion, resection of pancreatic tumors should be considered, even if the mass is suspicious for metastatic disease including recurrence of adrenocortical cancer.

### Consent

Written informed consent was obtained from the patient for publication of this case report and any accompanying images. A copy of the written consent is available for review by the Editor of this journal.

## Abbreviations

ACC: Adrenocortical carcinoma
ENSAT: European Network of the Study of Adrenal Tumours
DHEA-S: Dehydroepiandrosterone sulphate
SF-1: Steroidogenic factor 1
pan-ck: Pancreatic cytoceratine
